# Moving beyond multi-triazole to multi-fungicide resistance: Broader selection of drug resistance in the human fungal pathogen *Aspergillus fumigatus*

**DOI:** 10.1371/journal.ppat.1012851

**Published:** 2025-02-10

**Authors:** Spyros G. Kanellopoulos, Eveline Snelders

**Affiliations:** Laboratory of Genetics, Wageningen University & Research, Wageningen, the Netherlands; Duke University Medical Center, UNITED STATES OF AMERICA

## The need for triazoles: *Aspergillus fumigatus* treatment and antifungal resistance

**Fig 1:**
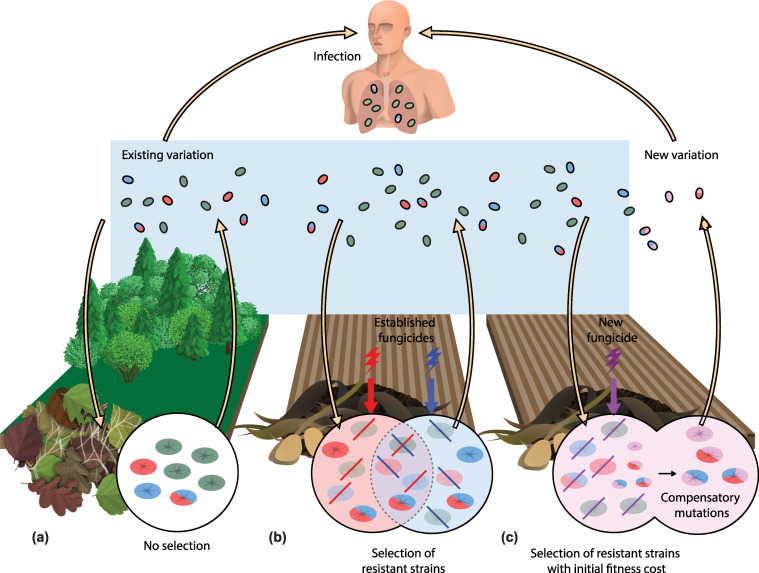
Model for the evolution of multi-fungicide resistance in *Aspergillus fumigatus*. **(a)** Conidia of *A*. *fumigatus* disperse through the air, colonizing natural areas without fungicides, such as parks and forests. In the absence of fungicides, all genotypes, resistant or sensitive, can propagate through airborne conidia. **(b)** In agricultural fields, the storage of decomposed organic matter allows growth of *A*. *fumigatus* and fungicide residues will select for resistant individuals, outcompeting fungicide-sensitive individuals. Considering that agricultural fungicides, including demethylation inhibitors (DMIs) are highly correlated with each other’s agricultural fungicide resistance, there is high selection pressure for multi-fungicide–resistant strains. **(c)** The introduction of a novel agricultural fungicide selects resistance from the standing variation of this population, potentially with an initial fitness cost in the presence of no fungicides. Nevertheless, strains with compensatory mutations will quickly be selected for, outcompete strains still with a fitness cost, and disperse via the air. This creates new variation of multi-fungicide–resistant strains, but this time with a broader fungicide resistance including the novel fungicide (figure design by Marc Maas).

*Aspergillus fumigatus* is a ubiquitous saprotrophic fungus that is commonly found growing in decaying plant matter where it recycles carbon and nitrogen [1]. *A*. *fumigatus* conidia can disperse very efficiently through the air and grow on many different natural substrates **(Fig 1A)**. Furthermore, due to its ubiquitous presence and daily inhalation, it is an important human fungal pathogen that poses a threat primarily to individuals with compromised immune systems, leading to several types of often fatal infections [2]. Among other diseases, *A*. *fumigatus* can cause chronic pulmonary aspergillosis (CPA) and invasive aspergillosis (IA). Although varied species of *Aspergillus* can be pathogenic, *A*. *fumigatus* is responsible for most cases of IA, one of the most severe forms of invasive fungal disease [2].

When treating aspergillus diseases, the first choice of antifungal drugs are triazoles that target the fungal ergosterol pathways [3]. By inhibiting lanosterol 14-α-demethylase encoded by the genes *cyp*51A and *cyp*51B, triazoles prevent the formation of ergosterol, a key component of fungal plasma membranes [3]. Triazole resistance in *A*. *fumigatus* was originally identified in isolates of chronic infections and was thought to be primarily the result of prolonged treatment with triazole antifungals, and is likely an evolutionary dead-end without further dispersal [4]. Most commonly resistance is correlated with variations in the target gene *cyp*51A [4]. More specifically, point mutations in the coding sequence and a tandem repeat (TR) of 34 or 46 bases in the promoter region of *cyp*51A (TR_34_/Leu98His; TR_46_/Tyr121Phe/Thr289Ala) result in alteration of the protein sequence and increased transcription and expression of CYP51A [5]. These variants are currently the predominant triazole resistance mechanisms in environmental and clinical isolates of *A*. *fumigatus*, but can vary depending on local epidemiology [6]. Therefore, multi-triazole-resistance in *A*. *fumigatus* challenges current antifungal treatment strategies.

## The core problem: Dual use of antifungal classes in medicine and agriculture

The dual use of chemicals in the environment and in the clinic can be an important factor in the development of resistant *A*. *fumigatus* infections in humans. The isolation of triazole-resistant *A*. *fumigatus* from environmental sources has been demonstrated to coincide with the prevalent use of triazole fungicides in agriculture [7,8]. Furthermore, triazole-resistant isolates have been shown to be genetically related between geographical regions and different types of environment, but cluster apart from triazole-sensitive isolates [9]. Resistance can be selected for during long-term clinical antifungal treatment or through the use of agricultural triazoles by selecting for resistance in decomposing organic matter and creating an environmental route for resistance selection [8]. Although *A*. *fumigatus* is not a plant pathogen and is therefore not an intended target for agricultural triazoles, many of these triazole compounds do show *in vitro* activity against *A*. *fumigatus* [7]. This pattern of unintended resistance selection is likely not limited to triazoles, but also to other agricultural fungicides.

The selection of resistant genotypes depends not only on the presence of fungicides in environmental settings, but also on conditions that favor the growth of the fungus, and ample time is needed for adaptation and competition to take place. Agricultural practices such as prolonged storage up to several months of plant waste before composting, create the favorable conditions mentioned for prolonged growth of *A*. *fumigatus* in the presence of fungicides **(Fig 1B)** [10].

## Broader than triazoles: Developing resistance to MBCs, SDHIs, and QoIs

Recently, *A*. *fumigatus* has received increased attention as an undesired off-target effect of agricultural triazoles. The interplay between *A*. *fumigatus* and agricultural environments, including both triazole and non-triazole fungicides, requires further investigation. How these agricultural niches shape the population structure of this fungus is a key question yet to be answered.

Although triazoles are commonly used in agriculture, other widely used fungicides, most notably methyl benzimidazole carbamates (MBCs), quinone outside inhibitors (QoIs), and succinate dehydrogenase inhibitors (SDHIs), have played a vital role in crop protection in the past decades. These fungicides, while effective in controlling plant pathogenic fungi, can also unintentionally be a risk for resistance selection for fungi residing in agricultural areas [11] when such compounds show activity against *A*. *fumigatus*. This seems to apply to *A*. *fumigatus*, which has been shown to harbor resistance alleles for these fungicides in a wide variety of countries [12–16]. Whole-genome sequencing not only supports a genetic connection among multi-fungicide-resistant strains, emphasizing a shared ancestry and reinforcing the relatively recent environmental origin of TR-based triazole-resistant *A*. *fumigatus* [9,17]. However, these studies also indicate that triazole-resistant isolates are much more likely to also exhibit resistance mutations to other types of fungicides such as MBCs, QoIs, and SDHIs than triazole-sensitive isolates. This suggests that the presence of a triazole resistance mutation is correlated with additional non-triazole antifungal resistance mechanisms, although the order in which different fungicide resistance variants were acquired remains unclear [12–16].

Agricultural fungicides can select fungal strains that exhibit resistance. Since residues of many different agricultural fungicides can simultaneously occur in organic waste material, particularly possible due to the multiple modes of action in some fungicide product formulations, multi-fungicide–resistant strains will have a competitive advantage in these niches. **(Fig 1B)**. Due to the broad use of multiple fungicide classes in agriculture, the multi-fungicide–resistant strains are likely to co-occur with triazole resistance. Therefore, the use of non-triazole fungicides to protect crops may enable continued selection of triazole-resistant *A*. *fumigatus* even in the absence of triazoles. Historically, the introduction of new fungicides in agriculture has always inevitably led to the development of resistance in fungi. **(Fig 1C)**. This can result in ongoing selection for the multi-fungicide–resistant strains that will, over time, accumulate with a broad range of resistance mechanisms to multiple fungicide classes. Therefore, in the case of dual use antifungal compounds, such as the case of Dihydroorotate dehydrogenase (DHODH) inhibitors [18], exposure of *A*. *fumigatus* to a novel agricultural fungicide can result in the selection of strains resistant to olorofim which is a novel clinical antifungal of the same class, which can jeopardize progress in the development of antifungal drugs.

## The cost of resistance: Multidrug resistance and its potential associated fitness in *A*. *fumigatus*

Antimicrobial drugs are often designed to inhibit molecules that are essential for a pathogen. This means that mutations that alter these molecules and confer resistance could result in a decline in fitness. The pathogen may reproduce more slowly, spread less frequently, and possibly result in reduced virulence. A fitness cost can also be caused by mutations that result in the overexpression of the target molecule or an efflux pump that is responsible for the extrusion of the fungicide, as this overexpression comes with an allocation of energy cost [11]. This indicates that multi-fungicide-resistant strains of *A*. *fumigatus* would initially be quickly outcompeted in environments without selection unless the fungus has overcome this cost of fitness. Since the multi-fungicide-resistant *A*. *fumigatus* does not seem to have a fitness cost [19], this could either mean that there are no fitness costs related to the multi-fungicide-resistance nature of *A*. *fumigatus* or that the organism has a way of mitigating these fitness costs.

Compensating mutations have been identified in various organisms, allowing drug-resistant mutants to restore their fitness cost partially or even fully [20]. Resistance and its fitness cost are an evolutionary process that has occurred in many different species. The phenomenon of resistance to antibiotics and antivirals in bacteria and viruses, respectively, is often associated with a fitness cost [21,22]. Additionally, for other classes of fungicides such as MBCs, QoIs, and SDHIs, resistance has been shown to incur fitness cost in various fungal plant pathogens [23], suggesting a potential parallel in *A*. *fumigatus*. On the contrary, MBC resistance has not been shown to directly affect plant pathogens’ growth and even enhances virulence in certain species [24], while temperature-dependent fitness costs have been found to occur due to trade-offs between the target site, impacting microtubule function. The fitness costs of QoIs and SDHIs vary across fungal species, while some SDHI-resistant mutants exhibit reduced sporulation and pathogenicity, others show minimal impact, highlighting the complexity of assessing fitness under various conditions [25]. Regarding *A*. *fumigatus*, a study comparing TR_34_ and TR_46_ *cyp*51A triazole-resistant *A*. *fumigatus* mutant strains compared to their parental strain found no detrimental fitness cost in competition assays conducted in the absence of triazoles [19]. The accumulation of multiple resistant variants in different genes could have a fitness cost that requires the strain to also possess compensatory mutations, to remain competitive with the rest of the population in its direct environment.

Investigating the existence of compensatory mutations in *A*. *fumigatus* may lead to a deeper understanding of the evolutionary processes imposed by fungicides on it. Traditionally, sporulation and colony diameter have been used to measure the fitness of resistant fungal plant pathogens, as well as the fitness in planta [26]. For *A*. *fumigatus*, there have not been well-established fitness assays that mimic what is happening in an agricultural setting where nutrition and oxygen availability are different and there is intra- and inter-species competition. Moreover, an assay such as a small-scale compost model that mimics the environment and focuses more on relative fitness between strains in direct competition could be utilized to enable better and more appropriate predictions for the environment.

## Looking further: Multiple paths for triazole resistance selection

The occurrence of multi-fungicide–resistant *A*. *fumigatus* in the environment enabling selection for triazole resistance in A. *fumigatus* does not only depend on selection pressure of agricultural triazoles but these strains will be co-selected by other agricultural fungicides to which it has obtained resistance as well **(Fig 1B)**. Currently, due to the broad variety and intensive use of fungicides in agriculture, multi-fungicide–resistant strains have an advantage growing on plant-degraded material exposed to multiple fungicides and will outcompete those that are not multi-fungicide resistant. Thus, the research on triazole resistance selection should not only focus on triazoles but also non-azole fungicides currently used in agriculture and the susceptibilities *A*. *fumigatus* displays to those fungicides.

The effects of resistance mutations and potential compensatory variants depend on each other’s presence, suggesting a beneficial epistatic interaction. Multi-fungicide–resistant strains that may have multiple sets of these resistant and compensatory variants could lose these beneficial epistatic interactions during sex, resulting in offspring with reduced fitness. Competition during growth in the environment could remove these low-fitness hybrid offspring, eventually reducing gene flow between multi-fungicide–sensitive and multi-fungicide–resistant strains that are present in different niches. This could be a plausible explanation for the clustering of resistant isolates separated from sensitive isolates [9,15,17].

The emergence of multi-fungicide–resistant *A*. *fumigatus* highlights the need to expand research beyond triazole resistance alone. Environmental selection pressure from various fungicides, coupled with potential fitness costs, underscores the complexity of evolutionary processes driving resistance selection in *A*. *fumigatus* and emphasizes the importance of comprehensive strategies to address this growing challenge.
